# Regulatory pathway analysis of coat color genes in Mongolian horses

**DOI:** 10.1186/s41065-017-0048-y

**Published:** 2017-09-29

**Authors:** Bei Li, Xiaolong He, Yiping Zhao, Dongyi Bai, Wunierfu Shiraigo, Qinan Zhao, Dugarjaviin Manglai

**Affiliations:** 1 College of Animal Science, Inner Mongolia Agricultural University, Huhhot, 010018 People’s Republic of China; 2 Inner Mongolia Academy of Agricultural and Animal Husbandry Sciences, Huhhot, 010031 People’s Republic of China

**Keywords:** Mongolian horse, RNA-Seq, Coat color, Pigmentation, Tyrosine

## Abstract

**Background:**

Studies on the molecular genetics of horse skin pigmentation have typically focused on very few genes and proteins. In this study, we used Illumina sequencing to determine the global gene expression profiles in horses with white-colored coats and those with black-colored coats, with the goal of identifying novel genes that could regulate horse coat color.

**Results:**

Genes encoding ribosomal-associated proteins were highly expressed in horse skin. We found a total of 231 unigenes that were differentially expressed between horses with white coats and horses with black coats; 119 were down-regulated, and 112 were up-regulated. Many of the up-regulated genes in black horses, such as genes related to tyrosine metabolism, may directly regulate dark coat color. Keratin genes, MIA family genes, fatty acid-related genes, and melanoma-associated genes were also differentially regulated, which suggests that they may play important roles in coat color formation.

**Conclusions:**

These findings show that the transcription profiles from white and black horse skin provide useful information to understand the genetics underlying the control of skin melanin synthesis in horses, which may enhance our knowledge of human skin diseases, such as melanoma and albinism.

## Background

Mammalian coat colors are determined by the quantities and distributions of melanin, which are dependent on the interaction between the genotype and the environment [1]. The most important pigments, including melanin and its derivatives, are synthesized in melanocytes by oxidation of tyrosine or tyrosine-related materials.

Mutation analyses have identified various genes that are involved in determining coat color in the horse [2]. Many of these genes regulate the expression and distribution of melanin, and their mutation can cause different phenotypes of the coat, skin, and eyes. Many of these gene mutations are situated within more than 60 loci that affect phenotype, and most are highly conserved in mammals, though the extent of their effect on pigment deposition varies [3]. Genes that commonly regulate skin and coat color in different mammalian species can be separated into 2 categories: one regulates the production, proliferation, or migration of different types of melanocytes, and the other affects pigment synthesis directly. Therefore, the formation of different skin and coat colors is determined by the regulation of genes that can change the progression/differentiation of melanocytes or the process of melanin synthesis [2].

At the cellular level, melanocytes put pigment granules into hair and skin cells; the presence and function of melanocytes therefore determine the amount, type, and character of pigmentation. Melanocytes originate along the neural crest, which also gives rise to the spinal cord and brain, and then migrate to the skin during embryogenesis [4]. The importance of this is that the pigmentary and nervous systems are closely allied in the embryo, and certain genes affect both.

Melanocytes can produce either pheomelanin or eumelanin as determined by a receptor on their surface, MC1R [5]. This receptor is activated by melanocyte-stimulating hormone, which is secreted by the pituitary gland. When activation occurs, the cells form eumelanin, while in the absence of activation, the cells form pheomelanin [6]. Whether the receptor becomes activated is determined by 2 factors: the presence or absence of melanocyte-stimulating hormone, and the presence or absence of cell surface receptors. Because melanocyte-stimulating hormone is consistently available to most cells in horses, regulation at the level of surface receptors is more important.

Previous studies to identify genes involved in skin pigmentation have focused on genetic polymorphisms. In the present study, we generated transcriptome profiles for horses with black or white skin utilizing high-throughput RNA deep-sequencing technology. Black and white skin from Mongolian horses was collected, and differentially expressed genes were identified by RNA-Seq, a high-throughput sequencing platform that allows for the detection and quantification of transcripts at low abundance, including novel transcript units [7]. The identification of genes for melanin production, distribution, and formation can provide a theoretical basis for the selection of skin traits during the selective breeding of horses. Additionally, increased understanding of the molecular mechanisms involved in skin pigmentation may have significance for other animals, including humans, in terms of skin-related diseases such as melanoma and albinism.

## Methods

### Animal selection

This investigation involved 2 Mongolian horses with black coat color (black1, black2) and 2 Mongolian horses with white coat color (white1, white2). All guidelines of the Institutional Animal Ethics Committee and the Animal Care Guidelines of the Inner Mongolia Agricultural University were followed when conducting experiments on the Mongolian horses.

### Feeding and management of animals

The black and white Mongolian horses were raised under equivalent conditions. All animals had free access to the same natural pasture. The animals were provided the same feeding regime for 6 months prior to skin collection.

### Sampling and excision biopsy

Skin biopsies were taken from the 4 Mongolian horses to analyze the enzymatic activity and metabolic status of melanocytes in the skin. The samples were collected in an exam room at the Inner Mongolia Agricultural University according to internal protocols and procedures. To achieve both sedation and analgesia, each animal received an intravenous bolus of 0.4 ml of detomidine hydrochloride per 100 kg of body weight. The inside of the hind legs underwent a wide cleaning and disinfection. After shaving to remove the hair, an excisional biopsy was carried out, and a small area of ≤1 cm^2^ was removed and stored in a tube with a sample protector for tissues. The wound was then immediately sutured with re-absorbent closures.

### RNA extraction

Total RNA was extracted using Trizol (TaKaRa) according to the manufacturer’s instructions, and its purity and integrity were assessed by 1% agarose gel electrophoresis. Subsequently, genomic DNA was removed by treating the RNA sample with RNase-free DNase I for 30 min at 37 °C.

### cDNA library construction and Illumina sequencing

To obtain poly (A) mRNA from the total RNA, oligo (dT) magnetic beads were used (Illumina). The RNA was broken down into short fragments by the addition of fragmentation buffer. These short fragments were then used as templates for first-strand cDNA synthesis with random hexamers and reverse transcriptase (Illumina). To synthesize the second strand of the cDNA, a solution of RNase H (Illumina), DNA polymerase I (Illumina), dNTPs, and buffer was used. The ends of the resulting double-stranded cDNA fragments were repaired, and adapters were ligated. The final version of the cDNA library was prepared from these products by purification and subsequent amplification by PCR. Using the Illumina Hiseq 2000 platform, the four prepared cDNA libraries were sequenced, resulting in 100-bp paired-end reads.

### Sequence preprocessing and functional annotation

The raw sequence data was processed using a Perl script developed in our lab; it removed the adapter sequences and filtered the low-quality reads and genes with *N* ≥ 10%. The resultant clean sequence data were mapped to the horse genome (*Equus caballus*) using TopHat2 without the discordant or mixed options. The reads that were uniquely mapped to the horse genome were analyzed to determine the approximate gene abundance, and gene expression levels were calculated by the reads per kilobase per million mapped (FPKM). Additionally, differentially expressed genes between the 2 Mongolian horses with black coat color and 2 Mongolian horses with white coat color were estimated by edgR using FPKM based on multiple significance tests. If |log2 (fold change)| > 1.4 and FDR < 0.05, then these genes were considered as differently expressed.

### Enrichment analysis

The functional annotation and pathway enrichment of genes that were differentially expressed between black- and white-skinned horses were performed using the online analysis tool DAVID (DAVID 6.7: https://david.ncifcrf.gov/tools.jsp), which is a program that manages the enriched Gene Ontology (GO) terms and Kyoto Encyclopedia of Genes and Genomes (KEGG) pathways that characterize genes. The differentially expressed genes (DEGs) were mapped to the GO database, and the hypergeometric test was utilized to determine which GO terms were significantly enriched among the DEGs against the background of the horse genome; GO terms were considered to be significantly enriched in the DEGs when they had corrected *p*-values <0.05. To determine which KEGG pathways, and therefore which complex biological behaviors, were enriched in the DEG data, a similar method was used (threshold: corrected *p*-value <0.05).

### Quantitative real time PCR (qRT-PCR) validation

Immediately after its removal, a piece of skin (approximately 0.5 cm^2^) was frozen in liquid nitrogen until needed for later qRT-PCR analysis. Its total RNA was obtained using Trizol (TaKaRa) according to the manufacturer’s protocol. After its extraction, the total RNA was dissolved in nuclease-free water. Approximately 0.5 μg of total RNA was used as a template for first-strand cDNA preparation using the PrimerScript RT reagent kit (TaKaRa) according to the manufacturer’s instructions. The synthesized cDNA was diluted to 0.1 μg/μl for analysis by qRT-PCR (Bio-Rad) using the SYBR Green Realtime PCR Master Mix (TaKaRa). The housekeeping gene GAPDH was selected as the control. To determine the relative levels of gene expression, the 2^−ΔCt^ (ΔCt = Ct_target gene_ − Ct_GAPDH_) method was used. ANOVA (using SAS software 9.0) was used to determine which genes were differentially expressed between black- and white-colored horse skins.

## Results

### Assembly and functional classification of unigenes from horse skin

**Fig. 1:**
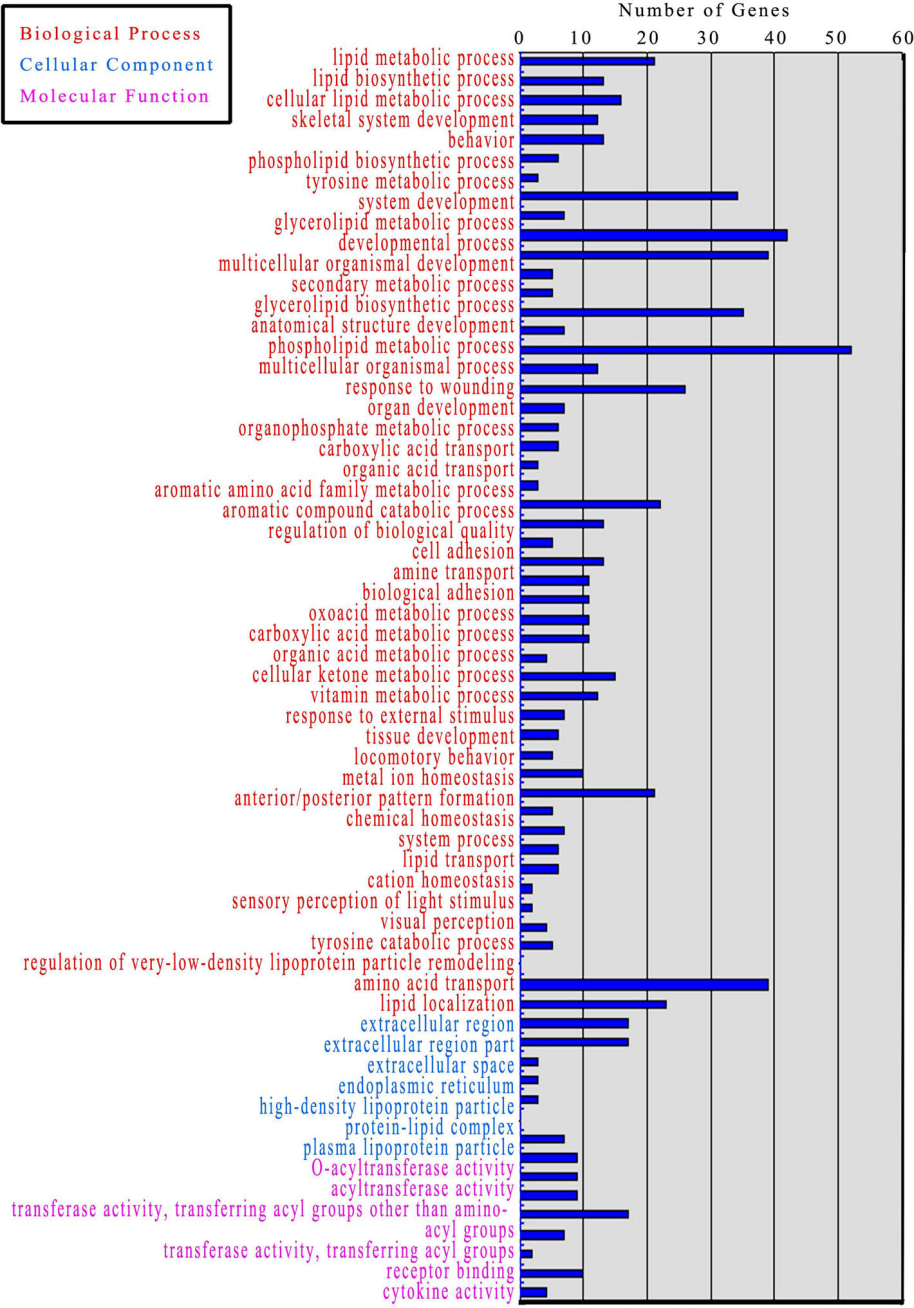
GO functional analysis of DEGs based on RNA-Seq data. The results can be separated into 3 main categories: “biological processes”, “cellular components”, and “molecular function”

A total of 24,301,563, 22,691,201, 19,423,074 and 20,465,943 pair-end reads were generated in the black1, black2, white1 and white2 libraries, respectively, after the raw reads were filtered from the skins of white and black horses. Of the 231 known genes, 202 were marked using GO analysis. This collection of genes was sorted into 63 classes using their putative functions as a basis for categorization. The largest collection of genes was sorted entirely by general function. Additionally, we annotated the known genes utilizing GO classification analysis and sorted them into 3 groups (74.6% were “biological processes,” 11.1% were “cellular components,” and 14.3% were “molecular function”) based on their presumed functions (Fig. 1).

### Highly expressed genes in horse skins

The keratin-family gene latherin (*LATH*) was the most highly expressed gene in horse skin (Table 1). Twenty-five of the 30 most highly expressed genes encoded ribosomal proteins; *Equus caballus* eukaryotic translation elongation factor 1 alpha 1 (EEF1A1) and three unknown genes were also highly expressed in the horse skin.

**Table 1:** The 30 genes highly expressed in horse skin

Gene	Black coat color FPKM	White coat color FPKM
LATH	72,811	20,843
LOC100053601	27,981	7759
RPL37	22,692	15,081
EEF1A1	15,280	16,186
RPS12	11,689	10,908
RPS27	10,792	9377
RPL23A	10,674	8141
RPL31	10,403	9728
RPL10	10,021	1180
RPL30	9785	7974
RPL35	8571	7940
RPS26	8320	7405
RPS3A	8190	8098
RPL13A	8138	7984
RPS19	8102	7883
RPL36A	7815	1615
RPS25	7764	6562
LOC100146711	7500	6102
LOC100630410	7423	5754
RPLP0	7281	7544
RPS20	7247	7066
RPS11	7117	6546
RPL26	7102	7405
RPS7	6983	6742
RPS23	6869	5602
RPS18	6865	5851
RPL35A	6761	5431
RPL17	6699	6305
RPL39	6606	6870
RPL9	6372	5806

### GO enrichment analysis of in genes that are differentially expressed in black- and white-colored skins

We assessed the 202 unigenes that were differentially expressed in black and white coat color horses by GO enrichment analysis. Among 97 known genes that were classified as “biological processes,” 15 genes were related to tyrosine metabolism; additionally, there were 58 known genes related to skin development. Among the 54 unigenes that were classified as “cellular components,” 17 genes were related to the endoplasmic reticulum; and among 41 known “molecular function” genes, 13 genes were related to acyltransferase activity and carboxylesterase activity (Table 2).

**Table 2:** GO ontology of differentially expressed genes of black and white horse skin

GO ontology	Cluster frequency		*P* value
	DEG	%	
Biological processes			
Lipid metabolic process	21	1.41	2.60E-05
Lipid biosynthetic process	13	0.87	2.70E-05
Cellular lipid metabolic process	16	1.07	5.74E-05
Skeletal system development	12	0.80	1.15E-04
Behavior	13	0.87	8.62E-04
Phospholipid biosynthetic process	6	0.40	2.04E-03
Tyrosine metabolic process	3	0.20	2.65E-03
System development	34	2.28	2.84E-03
Gycerolipid metabolic process	7	0.47	3.05E-03
developmental process	42	2.81	3.71E-03
Multicellular organismal development	39	2.61	4.03E-03
Secondary metabolic process	5	0.33	5.13E-03
Glycerolipid biosynthetic process	5	0.33	5.37E-03
Anatomical structure development	35	2.34	5.56E-03
Phospholipid metabolic process	7	0.47	6.62E-03
Multicellular organismal process	52	3.48	6.63 E-03
Response to wounding	12	0.80	6.97E-03
Organ development	26	1.74	8.40E-03
Organophosphate metabolic process	7	0.47	8.42E-03
Carboxylic acid transport	6	0.40	9.57E-03
Organic acid transport	6	0.40	9.84E-03
Aromatic amino acid family metabolic process	3	0.20	0.0119
Aromatic compound catabolic process	3	0.20	0.0131
Regulation of biological quality	22	1.47	0.0169
Cell adhesion	13	0.87	0.0201
Amine transport	5	0.33	0.0202
Biological adhesion	13	0.87	0.0203
Oxoacid metabolic process	11	0.74	0.0246
Carboxylic acid metabolic process	11	0.74	0.0246
Organic acid metabolic process	11	0.74	0.0257
Cellular ketone metabolic process	11	0.74	0.0276
Vitamin metabolic process	4	0.27	0.0282
Response to external stimulus	15	1.00	0.0300
Tissue development	12	0.80	0.0321
Locomotory behavior	7	0.47	0.0339
Metal ion homeostasis	6	0.40	0.0346
Anterior/posterior pattern formation	5	0.33	0.0349
Chemical homeostasis	10	0.67	0.0365
System process	21	1.41	0.0384
Lipid transport	5	0.33	0.0389
Cation homeostasis	7	0.47	0.0404
Sensory perception of light stimulus	6	0.40	0.0418
Visual perception	6	0.40	0.0418
Tyrosine catabolic process	2	0.13	0.0431
Regulation of very-low-density lipoprotein particle remodeling	2	0.13	0.0431
Amino acid transport	4	0.27	0.0448
Lipid localization	5	0.33	0.0496
Cellular component			
Extracellular region	39	2.61	8.82E-07
Extracellular region part	23	1.54	1.63E-05
Extracellular space	17	1.14	2.10E-04
Endoplasmic reticulum	17	1.14	6.86E-03
High-density lipoprotein particle	3	0.20	0.0186
Protein-lipid complex	3	0.20	0.0350
Plasma lipoprotein particle	3	0.20	0.0350
Molecular function			
O-acyltransferase activity	7	0.47	7.67E-07
Acyltransferase activity	9	0.60	2.76E-04
Transferase activity, transferring acyl groups other than amino-acyl groups	9	0.60	2.95E-04
Transferase activity, transferring acyl groups	9	0.60	3.83E-04
Receptor binding	17	1.14	2.77 E-03
Cytokine activity	7	0.47	5.80E-03
Hemoglobin binding	2	0.13	0.0248
Protein dimerization activity	10	0.67	0.0371
Carboxylesterase activity	4	0.27	0.0460

% = DGE/BGDGE × 100%; DEG represents the number of differentially expressed genes annotated to each GO ontology. BGDEG represents the number of all background differentially expressed genes annotated to each GO ontology

### KEGG enrichment of differentially expressed genes in black and white coat color skin

The differentially expressed genes were annotated in the KEGG database. Fourteen KEGG pathways were identified, including pathways associated with tyrosine metabolism (*DCT*, *TAT*, *HPD*), PPAR signaling (*ME1*, *APOA1*, *SCD*, *APOA5*), cell adhesion (*CLDN17*, *ITGAV*, *CD274*, *CNTN1*, *PDCD1LG2*), and ubiquinone and terpenoid-quinone biosynthesis (*TAT*, *HPD*) (Table 3).

**Table 3:** Enriched pathways of differentially expressed genes in black and white skin

Pathways	Differentially expressed genes^a^	KEGG unigenes^b^	*P* value	Pathway ID
PPAR signaling pathway	4	69	0.023	hsa03320
Cell adhesion molecules	5	143	0.028	hsa04514
Tyrosine metabolism	3	38	0.057	hsa00350
Ubiquinone and other terpenoid-quinone biosynthesis	2	11	0.060	hsa00130

^a^Differentially expressed genes in each pathway in the KEGG database

^b^Gene numbers in this pathway in the KEGG database

### Analysis of differentially expressed genes in black and white coat color skin

**Fig. 2:**
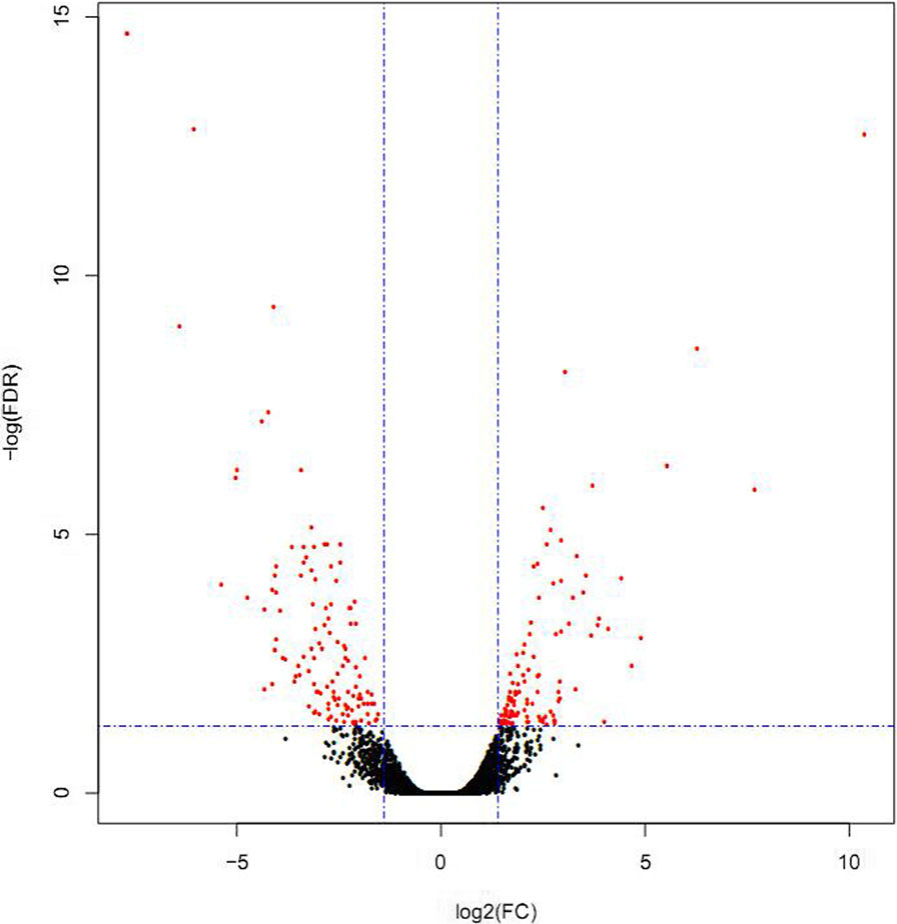
Comparison between gene expression levels of black and white horse skin libraries. The X and Y-axes show the mRNA expression levels in the 4 samples. The up-regulated and down-regulated genes are represented in red and black

When gene expression in white- and black-colored coats was compared, we found 231 genes that were differentially expressed; 119 DEGs were down-regulated in white coat color, and 112 DEGs were up-regulated (Fig. 2). The 20 genes with the most significantly different expressions are listed in Table 4; this list shows 10 up-regulated genes and 10 down-regulated genes.

**Table 4:** The top 10 up-regulated and down-regulated differentially expressed genes in white coat color vs. black coat color

Gene	Log_2_ (White FPKM/Black FPKM)	UP/DOWN	Function
MHCB3	10.379	up	MHC class I protein complex
CALCB	5.537	up	hormone activity
CPSF4L	4.888	up	metal ion binding
LOC100057688	4.685	up	protein binding
UNKNOW	3.833	up	oxidoreductase activity, acting on paired donors, with incorporation or reduction of molecular oxygen, reduced flavin or flavoprotein as 1 donor, and incorporation of 1 atom of oxygen
LOC100054146	3.707	up	nucleosome assembly
FOSB	3.674	up	sequence-specific DNA binding
CACNB4	3.548	up	protein binding
UNKNOW	3.506	up	protein binding
CHODL	3.327	up	carbohydrate binding
CPNE6	−7.670	down	protein binding
UNKNOW	−6.393	down	protein binding
LOC100629895	−6.058	down	mammaglobin-A-like
UNKNOW	−5.387	down	protein binding
C1QTNF6	−5.038	down	collagen trimer
NALCN	−4.743	down	cation channel activity
LOC100061216	−4.383	down	integral component of membrane
HBB	−4.327	down	oxygen transport
OTOR	−4.318	down	protein binding
UNKNOW	−4.229	down	keratin filament

### Verification of differential gene expression between horse skins using quantitative real-time PCR (qRT-PCR)

**Fig. 3:**
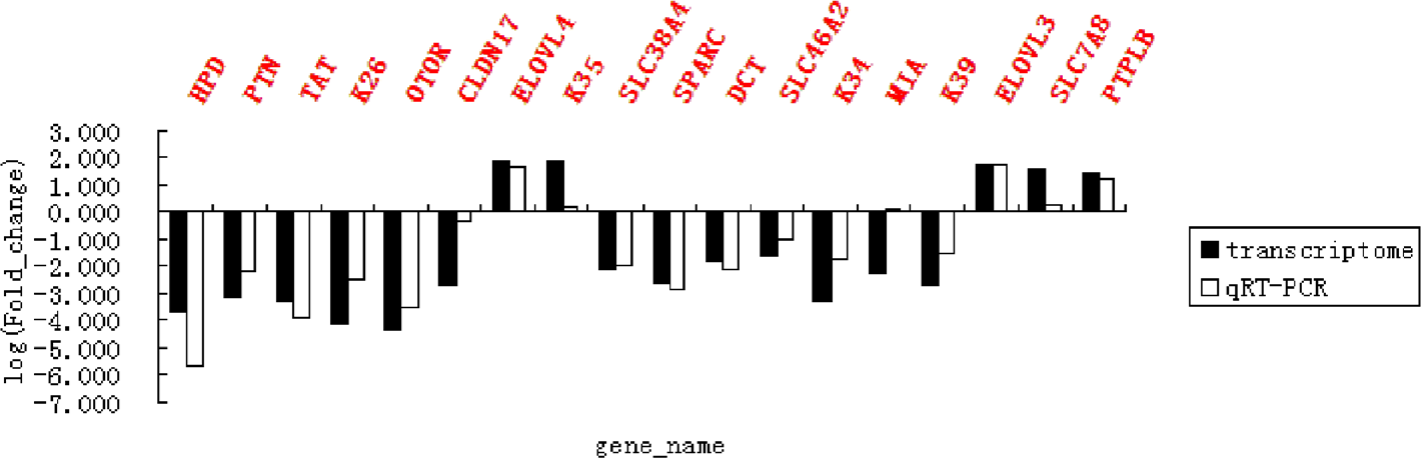
qRT-PCR validation of DEGs characterized by RNA-Seq. The results verify the differential expression of genes in black and white coat color skin, which is consistent with the RNA-Seq findings

To verify the differential expression of genes in black and white coat skin, we randomly selected 18 differentially expressed genes, *DCT*, *SLC38A4*, *K26*, *K34*, *K39*, *CLDN17*, *SPARC*, *SLC46A2*, *PTN*, *HPD*, *TAT*, *OTOR*, *K35*, *PTPLB*, *SLC7A8*, *MIA*, *ELOVL3* and *ELOV4*, to validate the expression patterns by qRT-PCR. For these 18 genes, the mRNA expression levels determined by qRT-PCR and RNA-Seq were significantly correlated (correlation coefficient = 0.825, *p* < 0.05), confirming that our RNA-Seq data was highly reproducible (Fig. 3).

## Discussion

The mechanisms of melanogenesis are intricate, and recent publications have furthered our understanding of melanin production in the skin [8, 9]. The interactions of several basic factors, most of which are from single genetic loci, are responsible for the various colors of most horses. With the development of advanced sequencing technology, skin and coat color genetics have been well studied [10]; however, most studies have focused on gene polymorphisms, whereas the impact of gene expression on coat color has not been well characterized.

The GO and KEGG pathway analyses indicated that the vast majority of the DEGs were related to “biological processes”. The pathways related to tyrosine metabolic and catabolic processes were of particular interest in our dataset. Tyrosine is a non-essential amino acid responsible for melanin production via its oxidation and polymerization [11].

**Fig. 4:**
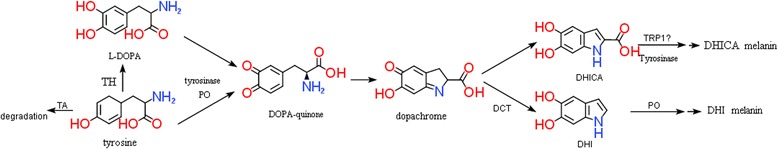
Common pathways of melanin synthesis in animals. Enzymes are abbreviated as follows: tyrosine aminotransferase, TA; tyrosine hydroxylase, TH; phenoloxidase, PO; dopachrome tautomerase, DCT; tyrosinase-related protein 1, TRP1; 5,6-dihydroxyindole, DHI; 5,6-dihydroxyindole-2-carboxylic acid, DHICA

To confirm the Illumina results, we performed qRT-PCR for specific genes. The *DCT*, *HPD*, and *TAT* genes were verified to be differentially expressed in white and black horses. These genes each participate in tyrosine metabolism. Dopachrome tautomerase (DCT) catalyzes the rearrangement of dopachrome to the carboxylated derivative DHICA [12]. The results of DCT on UV DNA damage and survival pathways in human melanocytic cells were determined by knockdown tests in melanoma cells, neonatal foreskin melanoblasts in monoculture, and co-cultures with human keratinocytes [13, 14]. DCT plays a major role in the coat color of cattle [15, 16] and rhesus macaques [17]. At the *DCT* locus, 3 mutations are known that affect pigmentation phenotypes. Guyonneau (2004) generated a knockout of the *Dct* gene in mice with effects restricted to pigment production and coat color [18]. As shown in Fig. 4, DCT is involved in regulating eumelanin and pheomelanin levels [19].

The Fe(II)-containing non-heme oxygenase 4-hydroxyphenylpyruvate dioxygenase (HPD) catalyzes the conversion of 4-hydroxyphenylpyruvate into homogentisate in the catabolism of tyrosine. HPD is linked to 1 of the oldest known inherited metabolic disorders, alkaptonuria, which is caused by low levels of homogentisate in the bloodstream. HPD is also directly linked to type III tyrosinemia [20]. When its concentration is low in the human body, high levels of tyrosine occur in the blood, which can cause mild mental retardation at birth and subsequent degradation in vision [21]. The liver enzyme tyrosine aminotransferase (TAT) catalyzes the transformation of tyrosine into 4-hydroxyphenylpyruvate [22]. It is the rate-limiting enzyme in the tyrosine catabolic pathway that also includes HPD [23]. Because the accumulation of tyrosine in blood causes toxic effects to tissues and organs [24], the breakdown of tyrosine by TAT is very significant for human health. Type II tyrosinemia is caused by a deficiency in TAT [25]. Thus, each of these differentially regulated genes has functions in important processes affected by tyrosine metabolism. These 3 genes were up-regulated in the tyrosine metabolism pathway in horses of black coat color, indicating that the *DCT*, *HPD*, and *TAT* genes affect the formation of dark coat color.

In our study, 4 keratin genes were indicated to be differentially expressed in black/white horse skin: *keratin 35* was up-regulated in white coat color skin, and *keratin 34*, *keratin 26*, and *keratin 39* were up-regulated in black coat color skin. Keratinocytes generate a large number of paracrine factors that affect the growth of pigment cells and their proliferation and behavior; changes in the expression of these factors affect melanocytosis via receptor-mediated signaling pathways [26]. This result suggests that the keratin family also affects dark coat color formation.

We also identified 2 differentially expressed genes from the melanoma inhibitory activity (MIA) family: MIA and otoraplin (OTOR). MIA has been shown to have growth-inhibitory activity on malignant melanoma cells in vitro [27, 28]. MIA is expressed and secreted by melanoma cells, but not melanocytes, and its mRNA levels parallel progressive malignancy of melanocytic tumors [29, 30]. In fact, increased MIA serum levels are considered to be a reliable tumor marker in detecting and monitoring metastatic disease and responses to therapy [31, 32]. OTOR is secreted via the Golgi apparatus and may function in cartilage development and maintenance. A frequent polymorphism in the translation start codon of this gene, potentially associated with alternative polyA sites, is associated with forms of deafness [33]. Gray horses are at an increased risk for melanoma, with 70–80% over the age of 15 presenting with melanomas [34]. It is possible that melanoma in light coat color horses is associated with the expression of *MIA* family genes.

Other genes identified as differentially expressed in our study include *PTN*, *RGS13*, and *SPARC*. The secreted heparin-binding protein pleiotrophin (PTN) has been shown to be involved in cell growth and differentiation [35]. Not much is known about the effects of PTN on skin pigmentation and melanocyte function. Transfection studies, however, have shown that PTN decreases melanogenesis through MITF degradation via Erk1/2 activation [36]. In vitro, the chemotaxis of B cells is controlled by the regulator of G-protein signaling 13 (RGS13); this control is thought to be carried out by increasing the GTPase activity of G_α_ proteins that are coupled to chemokine receptors [37]. RGS13 expression also reduces cAMP production [38], which plays an important role in melanoma even though genetic alterations in components of this pathway are not commonly found in melanomas [39, 40]. The incorporation of collagen into the skin is controlled by secreted protein acidic and rich in cysteine (SPARC) (or osteonectin or BM-40) [41]. SPARC has various roles that it carries out in cooperation with many extracellular matrix units: it behaves as a de-adhesive molecule, regulates cytokine and growth factor activities, and inhibits the cell cycle [42]. SPARC has also been shown to be strongly expressed in advanced primary and metastatic melanomas [43]. Although the molecular events responsible for their activity remain to be defined, the upregulation of *PTN*, *RGS13*, and *SPARC* in black color skin could explain the incidence of melanoma in light coat color horses.

True white horses of the kind used in this study have the dominant-negative W allele of the KIT gene [44, 45]. KIT encodes stem cell factor (steel factor) that is involved in stem cell differentiation and subsequent melanocytic migration [46]. Failure of melanocyte migration results in white markings on horses, including all-white coats. We did not find any differences in gene expression of KIT in the present study, though this could be due to the small sample size. However, similar global gene expression profiling of black and white sheep skin using Illumina sequencing [47] and transcriptome profiling of black and white rabbits [48] also did not find differential expression of KIT. Similar to our results, these studies did find up-regulated genes related to tyrosine metabolism and melanogenesis, including DCT in sheep, as well as keratin family genes. Zhang and coworkers [49] cataloged global gene expression profiles in Lueyang chickens with white versus black skin by Illumina2000 sequencing, and found differential expression of KIT. They also discovered up-regulation of tyrosine metabolism genes in black skinned chickens, but not changes in the expression of keratin family members. Overall, these results and those of the present study indicate that while there are many common differences in gene expression between white and black skinned vertebrates, they also vary considerably by species. More work is needed to determine the specific factors involved and their mechanisms.

## Conclusion

In this study, differentially expressed genes in horses with white and black coat colors were screened using high-throughput sequencing. The genes identified in this study may affect the formation of horse coat color directly or indirectly. Although the genes controlling horse coat color formation are not completely known, the transcription analysis presented in this study provides valuable information. For the first time, we present a collection of genes that are differentially expressed in horse skins of different colors. Some genes have not been described previously, and others are known, but they are likely to be involved in skin pigmentation and other physiological functions. The description of these differentially expressed genes will allow further study of the molecular regulation of horse coat color.
